# Variation of Serum Uric Acid Is Associated With Gut Microbiota in Patients With Diabetes Mellitus

**DOI:** 10.3389/fcimb.2021.761757

**Published:** 2022-01-18

**Authors:** Weifeng Zhang, Ting Wang, Ruixue Guo, Wen Cui, Wei Yu, Zhihui Wang, Yumin Jiang, Minghan Jiang, Xiaojie Wang, Chao Liu, Jing Xiao, Jin Shang, Xuejun Wen, Zhanzheng Zhao

**Affiliations:** ^1^ Department of Nephrology, The First Affiliated Hospital of Zhengzhou University, Zhengzhou, China; ^2^ Department of Emergency, The First Affiliated Hospital of Zhengzhou University, Zhengzhou, China; ^3^ Department of Traditional Chinese Medicine, Shandong University of Traditional Chinese Medicine, Jinan, China; ^4^ Department of Pharmacology, Shandong University School of Basic Medical Sciences, Jinan, China; ^5^ Shanghai Mobio Biomedical Technology Co., Ltd., Shanghai, China; ^6^ Department of Chemical and Life Science Engineering, Virginia Commonwealth University, Richmond, VA, United States

**Keywords:** uric acid, 16S rRNA, diabetes mellitus, metabolism, gut microbiota

## Abstract

Diabetes mellitus is a metabolic disease closely related to a disordered gut microbiome. Diabetic patients usually suffer from various metabolic disorders, such as increased serum uric acid levels. Although serum uric acid levels depend partially on intestine excretion, the relationship between uric acid and gut microbiome in diabetic patients remains unknown. We collected a total of 126 fecal samples from diabetic patients for 16S ribosomal RNA gene amplicon sequencing and recorded clinical data. We analyzed the correlation between clinical indicators and gut microbiota of diabetic patients using Spearman analysis. Since uric acid was the most prominent one, we classified diabetic patients based on their uric acid levels to find the microbiome associated with uric acid disturbance. We constructed Kyoto Encyclopedia of Genes and Genomes (KEGG) pathway profiles using Phylogenetic Investigation of Communities by Reconstruction of Unobserved States (PICRUSt) to identify variations between the different groups. Among all the clinical indicators, uric acid had the strongest correlation with gut microbiota. First, we divided the patients into three groups according to their uric acid levels. The two low uric acid groups were similar, while the elevated uric acid group had significant differences in gut microbiota and metabolic pathways. The elevated uric acid group had a significantly lower gut microbiota diversity. At the genus level, this group had remarkably higher *Escherichia–Shigella* amounts and notably lower *Faecalibacterium*, *Oscillospiraceae_UCG−002*, and *Oscillospiraceae_UCG−005* amounts. The gut microbiota of the high uric acid group was predicted to be enriched in metabolism, human diseases, and lipopolysaccharide biosynthesis. Since the two low uric acid groups were similar, we regrouped and matched the abnormal uric acid patients with normal uric acid patients. The differences in gut microbiota and metabolic pathways related to nucleotide metabolism became more significant. The serum uric acid levels were associated with gut microbiome changes. This might be related to uric acid metabolism by gut microbes. Our study indicates that targeting the gut microbiome could help manage elevated uric acid levels.

## Introduction

The high prevalence of high uric acid-related complications among diabetic patients has attracted more and more attention worldwide for its serious health consequences and huge economic burden (Chen et al., 2017). Uric acid metabolism is typical in diabetic patients (Sharaf El Din et al., 2017); high uric acid levels can increase their risk of cardiovascular and renal complications (Soltani et al., 2013; Xu et al., 2013) and aggravate insulin resistance (Hu et al., 2021). The gut microbiota is not only significantly related to diabetes but also its cause (Vangipurapu et al., 2020; Zhang et al., 2021). The gut microbiota participates in purine and uric acid metabolism (Henson, 2021). The kidneys excrete two-thirds of the human body’s uric acid, and the rest is mainly removed through the intestine (Richette and Bardin, 2010). Since hyperuricemia and diabetes are both metabolic diseases, we explored whether hyperuricemia in diabetic patients is also associated with gut microbiota changes. We aimed to deepen the understanding of diabetes-related metabolic disorders and provide new treatment targets for diabetes combined with elevated uric acid.

## Materials and Methods

### Ethics Statement

All patients included in this project provided written informed consent. The First Affiliated Hospital of Zhengzhou University Ethics Review Committee granted ethical approval for the study (2019-KY-361).

### Patient Selection

In this cross-sectional study, we collected clinical information and fecal samples of 126 diabetic patients admitted to the First Affiliated Hospital of Zhengzhou University between October 2018 and October 2019. **Table 1** displays basic information about the patients, including body mass index (BMI), clinical indicators, and whether they take metformin. The inclusion criteria were as follows: 1) typical diabetic symptoms (polyuria, polydipsia, and unexplained weight loss) and random blood glucose ≥11.1 mmol/L, 2) fasting blood glucose ≥7.0 mmol/L, 3) twice oral glucose tolerance test (OGTT ≥11.1 mmol/L). One of the above three items can be included. Individuals suffering from digestive system diseases or abnormal kidney function who had taken antibiotics in the 3 previous months or a recent infection history were excluded. We classified the included patients into three groups according to their diet (low-, medium-, and high-purine diet). High-purine diet: drinking alcohol or eating seafood/intestines at least once a week or the usual diet is mainly meat. Medium purine diet: balanced meat and vegetable diet. Low-purine diet: the daily diet is based on food with low purine content, such as rice noodles, vegetables, fruits, eggs, and milk.

**Table 1:** Characteristics of the subjects according to sUA tertile.

	E_UA	M_UA	R_UA	p-value
	(n = 42)	(n = 42)	(n = 42)	
Gender				0.012
Female	14 (33.3%)	17 (40.5%)	27 (64.3%)	
Male	28 (66.7%)	25 (59.5%)	15 (35.7%)	
BMI (kg/m^2^)	26.1 [24.7; 29.0]	24.4 [22.6; 26.1]	22.9 [20.7; 25.4]	<0.001
Age (years)	50.5 [41.2; 62.5]	56.0 [44.5; 64.5]	61.0 [50.2; 67.8]	0.091
UA [μmol/L]	384 (47.8)	281 (25.2)	184 (40.7)	<0.001
SBP [mmHg]	125 [120; 138]	135 [124; 147]	126 [116; 137]	0.136
DBP [mmHg]	79.5 [74.0; 92.2]	82.0 [75.0; 90.0]	77.5 [71.2; 82.0]	0.124
Neut [10^9^/L]	3.74 [2.88; 4.49]	3.39 [2.69; 4.51]	3.30 [2.57; 4.66]	0.716
Lymph [10^9^/L]	1.91 [1.52; 2.22]	1.73 [1.37; 2.21]	1.54 [1.34; 1.92]	0.067
Glu [mmol/L]	8.06 (3.85)	7.89 (5.59)	6.68 (6.53)	0.482
Urea [mmol/L]	5.00 [4.53; 6.49]	5.02 [4.15; 5.70]	5.08 [4.50; 6.29]	0.552
Scr [μmol/L]	66.0 [60.0; 80.5]	62.0 [53.1; 69.8]	57.0 [50.5; 63.8]	0.008
ALB [g/L]	41.8 (8.07)	42.6 (4.27)	39.7 (5.36)	0.075
TCHO [mmol/L]	4.70 [3.46; 5.61]	3.96 [3.56; 4.58]	3.72 [3.00; 4.72]	0.023
TG [mmol/L]	1.84 [1.40; 2.83]	1.86 [1.07; 2.50]	1.04 [0.76; 1.55]	<0.001
HDL [mmol/L]	0.96 [0.83; 1.14]	1.02 [0.91; 1.19]	1.08 [0.88; 1.45]	0.276
LDL [mmol/L]	2.94 [2.12; 3.58]	2.54 [2.09; 2.97]	2.14 [1.67; 2.69]	0.021
HbA1C (%)	7.72 [7.20; 9.54]	8.98 [7.20; 10.8]	8.24 [7.20; 10.5]	0.775
Metformin				0.909
Yes	21 (50.0%)	19 (45.2%)	20 (47.6%)	
No	21 (50.0%)	23 (54.8%)	22 (52.4%)	

E_UA, elevated uric acid; M_UA, moderate uric acid; R_UA, reduced uric acid; BMI, body mass index; UA, uric acid; SBP, systolic blood pressure; DBP, diastolic blood pressure; Neut, neutrophil; Lymph, lymphocyte; Glu, glucose; Scr, serum creatinine; ALB, serum albumin; TCHO, total cholesterol; TG, triglyceride; HDL, high-density lipoprotein; LDL, low-density lipoprotein.

### Sample Collection

Fecal samples from the 126 included patients were collected with a disposable fecal collection device. All samples were stored at −80°C within 2 h until further analysis.

### DNA Extraction and Gene Sequencing

Microbial genomic DNA was extracted from stool samples as described previously (Godon et al., 1997). After DNA quality inspection, the DNA fragments of each sample encoding the V3-V4 region of 16S ribosomal RNA (rRNA) were amplified by polymerase chain reaction (PCR). Samples were chemically lysed using buffer [4 M guanidine thiocyanate, 10% N-lauroyl sarcosine, 5% N-lauroyl sarcosine, 0.1 M phosphate buffer (pH 8.0)] followed by physical lysis through incubation at 70°C for 1 h and mechanical lysis by bead beating. DNA was extracted using an EZNA Stool DNA Kit (Omega Bio-tek, Inc., GA, USA) and stored at −20°C until further analysis. PCRs were run in an EasyCycler 96 PCR system (Analytik Jena Corp., AG) using the following program: 3 min at 95°C followed by 21 cycles of 0.5 min at 94°C (denaturation), 0.5 min at 58°C for annealing, and 0.5 min at 72°C (elongation), with a final extension at 72°C for 5 min. The Shanghai Mubai Company used the MiSeq platform (Illumina Inc., CA, USA) to perform 16S rRNA gene sequencing. We synchronized our standard operating procedures and performed nucleotide sequencing of all samples in one sequencing center to decrease the confounding effects of the technical means.

### Operational Taxonomic Unit Clustering and Taxonomy Annotation

We clustered quality-filtered sequences into unique sequences and quantified representative sequences in a descending order using UPARSE analysis (version 11 http://drive5.com/uparse/). Operational taxonomic unit (OTU) classification was performed according to the obtained sequences, and bioinformatic statistical analysis was usually conducted on OTUs with a similarity level of 97%. The relationships and differences between bacterial species were analyzed based on the classification results. We compared RDP Classifier (version 2.2 http://sourceforge.net/projects/rdp-classifier/) to obtain the taxonomic information corresponding to each OTU and perform taxonomic analysis on the OTU sequences.

### Bioinformatic Analysis

To minimize the difference in sequencing depth between different samples, we sampled the original abundance matrix 100 times at a sequencing depth of 10,000. The Ace/Chao index and Shannon/Simpson index were used to estimate OTU richness and bacterial diversity. A rarefaction curve was used to confirm whether the amount of sequencing data of a sample was reasonable. Shannon–Wiener curves reflected the microbial diversity of each sample with different sequencing quantities. Rank-abundance curves were used to explain species abundance and species uniformity. Multivariate statistical methods, principal component analysis (PCA), and principal coordinates analysis (PCoA) were used to assign samples to different groups. We used R software to make statistical analyses and graphs, including heat maps and Venn diagrams to show the differences in species composition. Linear discriminant analysis (LDA) effect size (Segata et al., 2011) (LEfSe, version 1.0.8) was used to detect the abundance of differences at the taxon level. Taxa were shown if LDA values >2.0 with a p-value <0.05. The Phylogenetic Investigation of Communities by Reconstruction of Unobserved States (PICRUSt, version 1.1.4) was applied to predict the microbial functional profiles from the respective 16S taxonomic profiles (Langille et al., 2013). Next, R software was used to analyze the taxonomic and functional profiles.

### Statistical Analysis

**Figure 1:**
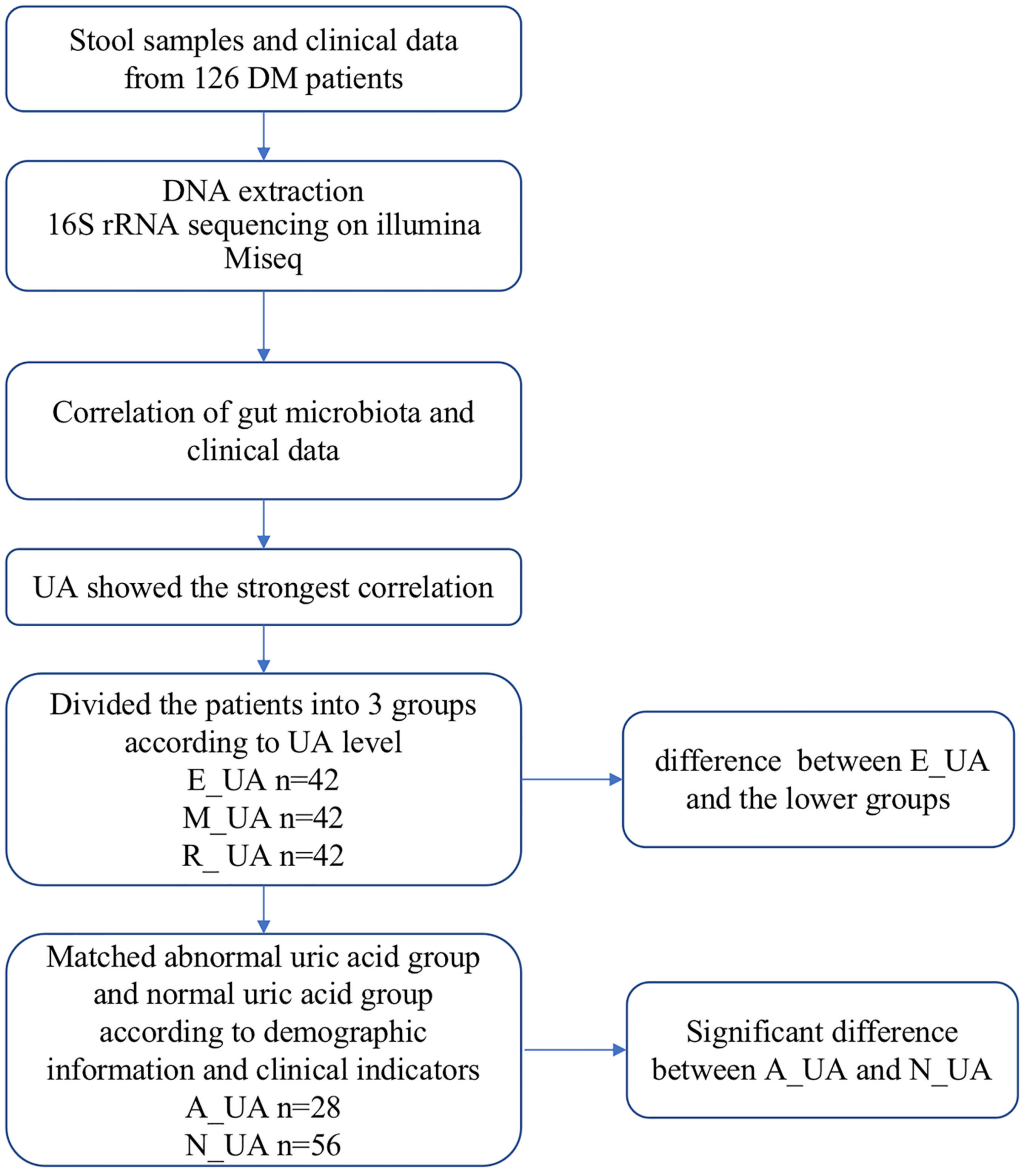
Study design and flow diagram. DM, diabetes mellitus; UA, uric acid; E_UA, elevated uric acid; M_UA, moderate uric acid; R_UA, reduced uric acid; A_UA, abnormal uric acid; N_UA, normal uric acid. Matching indicators include uric acid, systolic blood pressure, diastolic blood pressure, neutrophils, lymphocytes, glucose, serum creatinine, serum albumin, total cholesterol, triglycerides, high-density lipoprotein, and low-density lipoprotein.

We assessed the potential linear relationship between the gut microbiota and clinical indicators of diabetic patients using Spearman’s correlation analysis. We calculated the p-value to confirm the relationship between gut microbiota and uric acid. We used the propensity scoring method to match diabetic patients 1:2 according to their uric acid level using R software and adjust a series of demographic and clinical indicators with potential confounding effects such as age, gender, and blood lipids (**Figure 1** and **Table 2**). The data in the table were presented as mean (standard deviation). Unpaired Student’s *t*-test or Mann–Whitney U test were used to compare pairs of groups. The statistical significance of multigroup was assessed using one-way analysis of variance (ANOVA) with the Bonferroni *post-hoc* test. Random forest analysis was used to find the differences in key flora. p < 0.05 was considered statistically significant.

**Table 2:** Population characteristics of 1:2 matching between normal uric acid and abnormal uric acid group.

	A_UA (n = 28)	N_UA (n = 56)	p-value
Gender			0.469
Female	8 (28.6%)	22 (39.3%)	
Male	20 (71.4%)	34 (60.7%)	
Diet			0.541
L-purine	2	3	
M-purine	14	26	
H-purine	5	5	
Age (years)	50.3 (16.7)	53.0 (16.2)	0.481
SBP [mmHg]	127 [123; 140]	134 [120; 141]	0.977
DBP [mmHg]	84.2 (12.4)	81.1 (10.7)	0.254
Glu [mmol/L]	8.68 [6.69; 9.54]	8.00 [5.60; 10.3]	0.931
Neut [10^9^/L]	2.78 [0.00; 3.76]	2.84 [0.00; 4.27]	0.4
Lymph [10^9^/L]	1.89 (0.64)	1.91 (0.62)	0.889
Urea [mmol/L]	5.29 (1.75)	5.33 (1.62)	0.914
UA [μmol/L]	407 (41.4)	268 (55.3)	<0.001
Scr [μmol/L]	69.8 (15.7)	65.9 (16.6)	0.301
ALB [g/L]	43.3 [40.5; 44.6]	41.4 [39.1; 44.2]	0.329
TCHO [mmol/L]	4.92 (1.82)	4.36 (1.08)	0.143
TG [mmol/L]	2.85 (2.51)	2.03 (1.22)	0.112
HDL [mmol/L]	0.95 [0.80; 1.16]	1.03 [0.89; 1.31]	0.222
LDL [mmol/L]	3.05 [2.17; 3.56]	2.59 [2.06; 2.96]	0.068
Hb [g/L]	137 [129; 145]	134 [126; 144]	0.544

A_UA, abnormal uric acid; N_UA, normal uric acid; UA, uric acid; SBP, systolic blood pressure; DBP, diastolic blood pressure; Neut, neutrophil; Lymph, lymphocyte; Glu, glucose; Scr, serum creatinine; ALB, serum albumin; TCHO, total cholesterol; TG, triglyceride; HDL, high-density lipoprotein; LDL, low-density lipoprotein; Hb, hemoglobin.

## Results

### Correlation Between Gut Microbiota and Clinical Indicators

**Figure 2:**
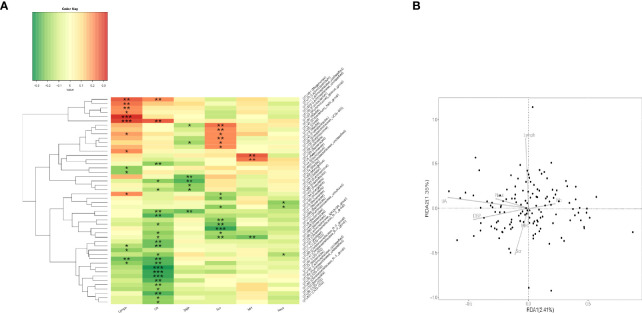
Correlation analysis of gut microbiota and clinical indicators. **(A)** Heatmap of Spearman rank correlation analysis between gut microbiota and clinical indicators such as UA, Scr, Lymph, and so on. *p < 0.05; **p < 0.01; ***p < 0.001. **(B)** The quadrants of the arrows indicate the positive and negative correlations between the corresponding clinical indicators and the gut microbiota. The longer the connection, the greater the correlation, and *vice versa*. The smaller the angle, the higher the correlation. Lymph, lymphocyte; UA, uric acid; DBP, diastolic blood pressure; Scr, serum creatinine; MH, metformin history; Neut, neutrophils.

The association between gut microbiota and clinical indicators such as uric acid, blood lipids, and creatinine was assessed by Spearman correlation analysis (**Figure 2**). Uric acid was significantly negatively correlated with multiple bacteria. To analyze their relationship and find key microbes in uric acid-disordered patients, we first divided patients into three groups according to their uric acid levels [Reduced uric acid group (R_UA), Moderate uric acid group (M_UA), and Elevated uric acid group (E_UA)] (**Figure 1**).

### Baseline Characteristics of the Three Uric Acid Level Groups

**Table 1** displays the BMI, clinical characteristics, and the status of taking metformin of the participants according to serum uric acid tertile. We can find that there is no statistical difference in the status of taking metformin among the three groups. We stratified the patients into three serum uric acid level groups with cutoff values of <240 μmol/L, 240–325 μmol/L, and >325 μmol/L.

### Richness and Diversity of Gut Microbiome Changed in High Uric Acid Patients

**Figure 3:**
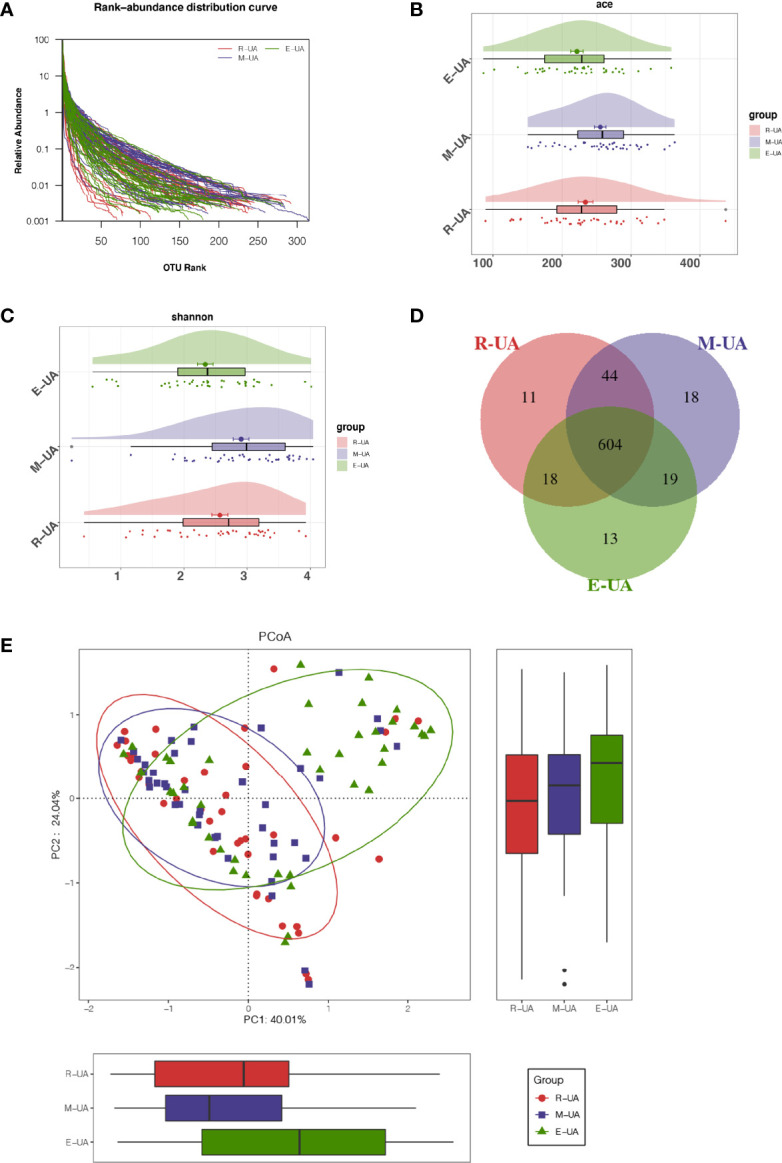
Decreased bacterial diversity in E_UA. **(A)** Rank-abundance curves indicated that the amount of sequencing data is reasonable. Bacterial richness and diversity (including richness and evenness) were assessed by Ace index (p = 0.028; **B**) and Shannon index (p = 0.003; **C**). **(D)** Venn diagram showed that there were 13 unique operational taxonomic units (OTUs) in E_UA group and 604 shared OTUs among the three groups. **(E)** Beta diversity visualized the dissimilarity of microbial community among samples using principal coordinates analysis (PCoA) based on unweighted UniFrac algorithum. E_UA, elevated uric acid.

We analyzed the gut microbial diversity of the R_UA, M_UA, and E_UA groups. The species accumulation curves (**Figure 3A**) indicated that the amount of sequencing data was reasonable and sufficient. The Venn diagram shows the similarities and differences between the bacterial communities of the three groups (**Figure 3D**). The Ace index indicated gut microbiota richness at the OTU level (**Figure 3B**). The R_UA, M_UA, and E_UA groups had Ace indices of 234.10, 255.34, and 221.62, respectively (p = 0.028). Meanwhile, the Shannon index reflected community diversity (**Figure 3C**). The Shannon indices of R_UA, M_UA, and E_UA groups were 2.57, 2.90, and 2.34, respectively (p = 0.003). The M_UA group had the highest abundance and diversity. The PCoA based on OTU levels showed that the R_UA and M_UA groups had similar community compositions, while that of the E_UA group differed (**Figure 3E**).

### Patients With Elevated Uric Acid Levels Had a Different Gut Microbiome Composition

**Figure 4:**
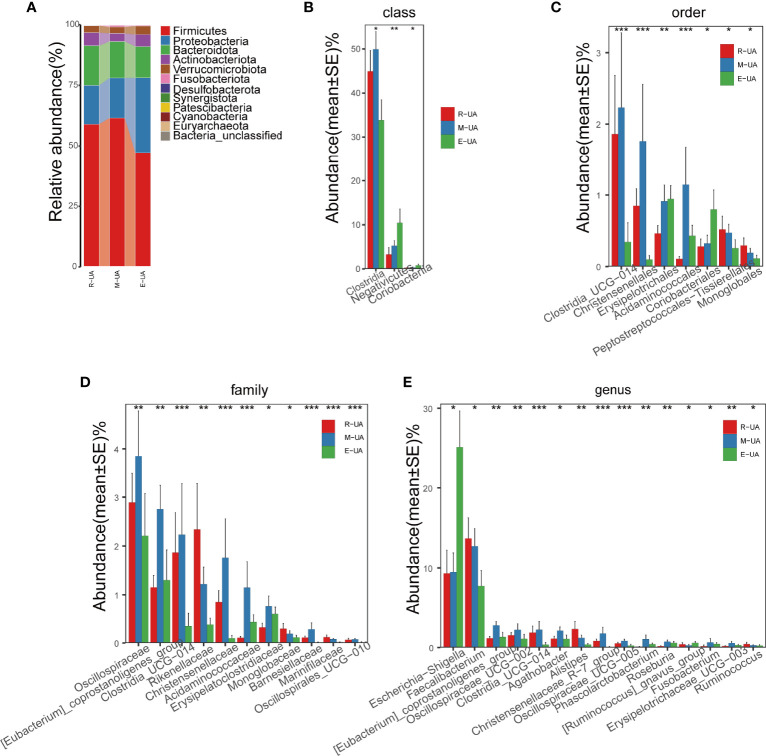
Composition of the microbial communities in the R_UA, M_UA, and E_UA groups. **(A)** R_UA and M_UA had similar average abundance at the phylum level, but *Firmicutes* and *Bacteroides* of E_UA were lower than those of the above two groups. **(B–E)** Relative abundance were significantly different between E_UA and the other two groups. R_UA, reduced uric acid; M_UA, moderate uric acid; E_UA, elevated uric acid. *p < 0.05, **p < 0.01, ***p < 0.001.

The R_UA and M_UA groups had similar average abundances at the phylum level, but the E_UA had lower *Firmicutes* and *Bacteroidetes* richness than that in the other two groups (**Figure 4A**). At the class level, the E_UA group had remarkably less *Clostridia* than the other groups, but more *Negativicutes* and *Coriobacteriia* (**Figure 4B**). At the order level, the E_UA group had much fewer *Clostridia_UCG−014* and *Christensenellales* but more *Coriobacteriales* (**Figure 4C**). At the family level, the E_UA group displayed less *Oscillospiraceae*, *Clostridia_UCG−014*, and *Christensenellaceae* (which belong to the *Clostridia* order) (**Figure 4D**). Finally, at the genus level, the E_UA group had high *Escherichia–Shigella* levels and low *Faecalibacterium* levels (**Figure 4E**).

### Functional Alteration of Gut Microbiota in High Uric Acid Patients

**Figure 5:**
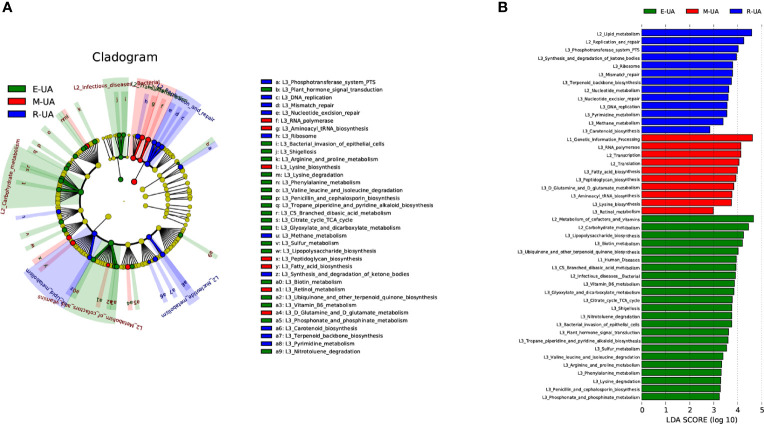
Microbiota-associated functional changes in the three groups. Distribution **(A)** and comparison **(B)** of Kyoto Encyclopedia of Genes and Genomes (KEGG) metabolic pathways among the three groups were shown at the taxonomic level using LEfSe analysis. LEfSe, linear discriminant analysis (LDA) effect size.

PICRUSt was used to identify differences in the Kyoto Encyclopedia of Genes and Genomes (KEGG) pathways of the three groups (**Figure 5A**). Among the six major metabolic pathways, metabolism was predicted to be more active in the R_UA group, and genetic information processing was more active in the M_UA group. Human disease was significantly increased in the E_UA group (**Figure 5B**). Functional categories including carbohydrate metabolism (glyoxylate and dicarboxylate, branched dibasic acid metabolism, and citrate cycle), amino acid metabolism (lysine, phenylalanine, arginine, and proline metabolism), cofactors and vitamins (vitamin B6 and biotin metabolism), and lipopolysaccharide biosynthesis showed higher levels in the E_UA group.

### Validation of Gut Dysbiosis Using New Grouping Cohort

Since the E_UA group had a significantly different gut microbiota from the other two groups, we chose to rearrange the groups. A reasonable explanation is that more significant changes in gut microbiota may occur when uric acid levels rise and harm the body. Therefore, we selected patients with uric acid levels above the normal range and matched them with patients whose uric acid level was within the normal range. After removing five participants with low uric acid levels, we matched abnormal uric acid (A_UA, n = 28) and normal uric acid (N_UA, n = 56) patients according to gender, age, blood lipids, and other clinical indicators. **Table 2** shows the grouping data. We divided the patients into low-, medium-, and high-purine diet groups. Since we conducted a post-event follow-up to know the patients’ diet, some did not reply. Among the 84 people in the two matched groups, 55 communicated their diet information. The specific values are shown in **Table 2**.

**Figure 6:**
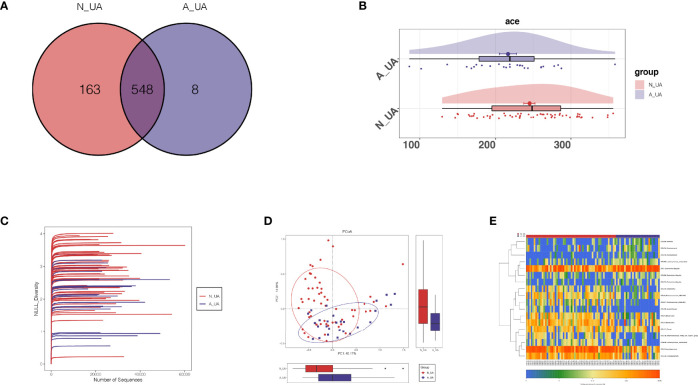
The taxonomic and functional differences between the two groups in the subgroup analysis. **(A)** Shannon–Wiener curves showing estimated operational taxonomic unit (OTU) richness basically approached saturation in all samples, and the microbial diversity was lower in the A_UA group. **(B)** Venn diagrams showing OTU distribution in different groups. Ace index (p = 0.023; **C**) showed that the richness of bacteria was lower in the A_UA group. **(D)** The β-diversity was visualized among the two groups in way of principal coordinates analysis (PCoA). **(E)** Heatmap showed 20 significantly different key OTUs between N_UA and A_UA.

**Figure 7:**
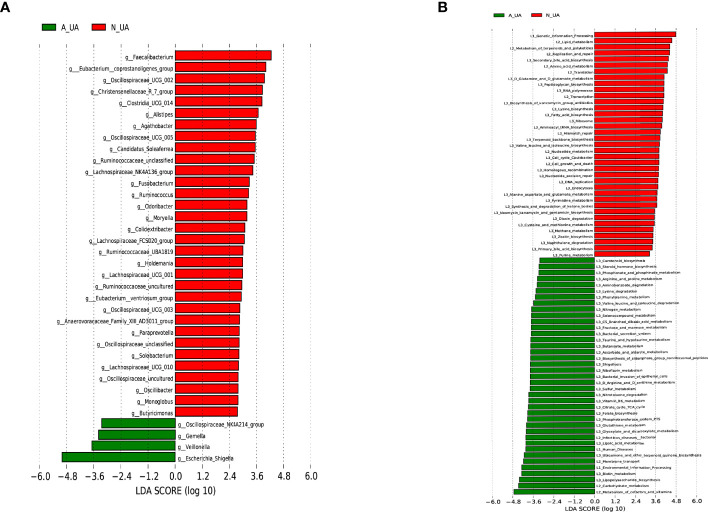
Linear discriminant analysis (LDA) effect size (LEfSe) analysis of microbial profiles and metabolic pathways. **(A)** LEfSe analysis of microbial profiles between N_UA and A_UA at the genus levels. **(B)** LEfSe analysis showing different metabolic pathways between N_UA and A_UA. N_UA, normal uric acid; A_UA, abnormal uric acid.

We found a total of 556 OTUs in the A_UA group and 711 in the N_UA group (**Figure 6A**). By calculating the alpha diversity index, we observed that the A_UA group had a lower OTU richness than that in the N_UA group (Ace indices: A_UA = 216.39, N_UA = 244.89, p = 0.048; Chao indices: A_UA = 205.58, N_UA = 242.29, p = 0.017) and diversity (Shannon indices: A_UA = 2.13, N_UA = 2.79, p = 0.001) (**Figures 6B**, **S1A, B**). The Shannon–Wiener curve (**Figure 6C**) indicated that the number of samples was sufficient, and the rank-abundance curve (**Figure S1C**) showed that the OTU spectrum had high abundance and was evenly distributed in the N_UA group. The PCoA revealed a marked difference between the A_UA and N_UA groups’ microbial communities (**Figure 6D**). The non-metric multidimensional scaling (NMDS) analysis (**Figure S1D**) based on Bray–Curtis distance or unweighted UniFrac dissimilarity yielded similar results. The heatmap showed 18 key OTUs with significantly different relative abundances between the two groups (**Figure 6E**). The LEfSe analysis showed that the gut microbes were significantly different at the genus level (**Figure 7A**).

Subgrouping revealed similar differences in intestinal bacteria. Consistent with the results of the three classification groups, the A_UA had a lower *Firmicutes* amount at the phylum level (**Figure S2A**). At the class level, the A_UA group had considerably fewer *Clostridia* (**Figure S2B**). At the order level, the A_UA group had relatively fewer *Oscillospirales*, *Clostridia_UCG−014*, and *Christensenellales* (**Figure S2C**). At the family level, *Ruminococcaceae*, *Oscillospiraceae*, *Prevotellaceae*, and *[Eubacterium]_coprostanoligenes*_group amounts were lower in the A_UA group (**Figure S2D**). At the genus level, the A_UA group had significantly more *Escherichia–Shigella* and fewer *Faecalibacterium*, *Oscillospiraceae_UCG−002*, and *Oscillospiraceae_UCG−005* (**Figure S2E**). Generally, the reduced bacteria were almost the same in the A_UA and E_UA groups, confirming their role in uric acid metabolism. At the genus level, *Escherichia–Shigella* was significantly enriched in both the A_UA and E_UA groups.

Besides, even though both groupings revealed differences in the same microbiota, the second grouping yielded more significant differences. In particular, at the genus level, the statistical differences in *Escherichia–Shigella* (p-values for the first and second grouping: 0.014 vs. 6.97 × 10^−5^), *Faecalibacterium* (p-values for first and second grouping: 0.050 vs. 0.003), and *[Eubacterium]_coprostanoligenes*_group (p-values for the first and second grouping: 0.003 vs. 4.73 × 10^−4^) were more significant. After regrouping, the obviously enriched metabolic pathways in A_UA were basically the same as those in E_UA, such as carbohydrate metabolism, cofactors and vitamins, and lipopolysaccharide biosynthesis. However, nucleotide metabolisms, including purine metabolism and pyrimidine metabolism, were active in the N_UA group (**Figure 7B**).

To confirm whether the differences observed in the E_UA group were associated with uric acid and diabetes or uric acid alone, we compared the gut microbiota of healthy controls and diabetic patients with high uric acid levels. We found that the latter had higher *Escherichia–Shigella* and lower *Faecalibacterium* amounts at the genus level (**Figure S3**).

## Discussion

After analyzing the correlation between the gut microbiota of diabetic patients and a series of clinical indicators such as uric acid and blood lipids, we found that the correlation between uric acid and gut microbiota was the most significant. Previous reports showed that uric acid is an independent risk factor for the complications of type 1 diabetes (Pilemann-Lyberg et al., 2019). To further explore whether gut microbiota may affect the diabetes progression by regulating uric acid, we divided diabetic patients into different groups according to their uric acid levels. Our research revealed a significant difference between the group with the highest uric acid levels and the other two groups. Rearranging the groups revealed an even more significant gut microbia diversity and abundance difference between the patients with normal and abnormal uric acid levels.

We first grouped the diabetic patients according to their median uric acid levels but found no significant difference in gut microbiota. We then grouped the patients by tertiles of uric acid levels and found that the two low uric acid groups had similar microbiota diversity and abundance, while the high uric acid group stood out. We speculated that the uric acid levels of the two low-level groups were within the normal range. This hypothesis implies that normal uric acid levels do not affect the gut microbiota of diabetic patients. To confirm this hypothesis, we grouped again and matched the diabetic patients with abnormal uric acid levels and those with normal uric acid levels. The new gut microbiota analysis revealed marked differences. We also grouped according to the international hyperuricemia standard (male uric acid ≥420 μmol/L, female uric acid ≥360 μmol/L), and the differential bacteria and metabolic pathways obtained were consistent with the grouping mentioned above (**Figure S4**). Through multiple groupings, our research revealed that elevated uric acid levels and gut microbiota changes are synergistic. It may be because when uric acid is within the normal range, gut microbiota compensates for its regulation. However, disturbed microbiota cannot regulate uric acid levels, which increase. Besides, high uric acid levels could affect the gut microbiota. The causal relationship needs to be verified by further experiments. However, the fluctuation of uric acid within the normal range is not relevant to the change in gut microbiota. In contrast, abnormal uric acid is associated with gut microbiota disturbance, which is enough to explain the regulation and compensation effect of gut microbiota on uric acid metabolism.

Previous studies identified specific gut microbial community changes associated with the presence of type 2 diabetes (Vangipurapu et al., 2020). Nevertheless, many studies regarded diabetes as a single predictor, neglecting the influence of various variables and complications in patients that may affect the gut microbiota. Previous studies have shown that diabetic patients and healthy people have different gut microbiota, but we do not know which clinical indicators associated with gut microbiota promote diabetes progression. This study explored the relationship between serum uric acid and gut microbiota of diabetic patients while controlling confounding factors including age, gender, blood lipids, and kidney function. Our results indicate that studies linking gut microbiota differences with type 2 diabetes need to consider uric acid levels as a potential confounding factor.

*Escherichia–Shigella* were overrepresented in the high uric acid group. Xi et al. (2000) demonstrated that these bacteria could secrete xanthine deaminase, which can convert hypoxanthine and xanthine into uric acid. In line with our findings, Mendez-Salazar et al. (2021) also revealed *Escherichia–Shigella* enrichment in gout patients. Moreover, *Escherichia–Shigella* enrichment has been found in other diseases. Therefore, many scholars believed that the *Proteobacteria* enrichment, including *Escherichia–Shigella*, was a characteristic of intestinal homeostasis imbalance (Guo et al., 2016). Thus, artificially adjusting the number and proportion of the bacteria may be the key to improving our intestinal health.

Intestinal dysbiosis and decreased short-chain fatty acid (SCFA)-producing bacteria are common in metabolic disorders, including diabetes and obesity (Candela et al., 2016; Ma et al., 2020; Yang et al., 2020). Hartwich et al. (2012) confirmed that *Clostridium cylindrosporum*, belonging to *Clostridiaceae*, is an anaerobic homologous SCFA-producing bacterium that can use purines such as uric acid as its only carbon, nitrogen, and energy source. The high-uric acid groups had significantly lower amounts of various bacteria belonging to the *Clostridia* at the genus level. The abnormal uric acid group had significantly lower amounts of the butyrate-producing *Faecalibacterium*, belonging to *Clostridia*, at the genus level. Therefore, we speculate that *C. cylindrosporum* and *Faecalibacterium* play a pivotal role in uric acid metabolism.

Previous studies showed that intestinal butyric acid amounts were related to uric acid metabolism in humans. Thus, butyric acid was expected to become another important treatment for elevated uric acid (Hamer et al., 2009). However, butyric acid has an unpleasant smell and is easily decomposed by oral supplementation (Shashni et al., 2021), while *Clostridium*, as an intestinal microbial preparation, is not affected by gastric acid and bile acid. It can therefore produce butyric acid by fermenting dietary fiber in the intestine (Dwidar et al., 2012). There is no doubt that this bacterium has a wide range of clinical application prospects.

In the normal uric acid group, besides *Faecalibacterium*, *[Eubacterium]_coprostanoligenes_group* was most enriched. This bacterium not only produces butyric acid but is also a key hub of fecal microbes for high-fat dieters, mainly through sphingosine to affect blood lipid metabolism (Wei et al., 2021). In animal experiments, regulating butyrate-producing bacteria reduced gouty arthritis caused by increased uric acid (Wen et al., 2020). Furthermore, chlorogenic acid supplementation can increase the relative abundance of intestinal SCFA-producing bacteria and reverse the purine metabolism of the intestinal microbiota (Zhou et al., 2021). The above studies all show that regulating butyrate-producing bacteria can improve purine metabolism.

However, the mean ages of the two groups in **Table 2** are 50.3 (16.7) and 53.0 (16.2). Although there is no statistical difference, this age span is relatively large. To explore the influence of age on the gut microbiota of these two groups, we divided the study population into three subgroups by age (<40, 40–55, and ≥55) and compared these subgroups (**Figure S5**). We found that in the two younger groups (ages <55), the significantly different bacteria are consistent with the above results. Namely, diabetic patients with high uric acid levels had high *Escherichia–Shigella* amounts and low *Faecalibacterium* amounts. However, in the older group (age ≥55), *Faecalibacterium* is not statistically different. We think that this may be due to the small sample size after multilevel grouping. Meanwhile, studies have shown that *Faecalibacterium* is reduced in healthy older adults (De Filippis et al., 2020), which may explain why the difference between the two groups is not statistically significant.

Vich Vila et al. (2020) studied the effects of some commonly used drugs on gut microbiota and showed that proton pump inhibitors, metformin, antibiotics, and laxatives strongly correlate with gut microbiota changes. The patients included in our study had no gastrointestinal diseases, no proton pump inhibitors, and no antibiotics and laxatives in the past 3 months. Therefore, we collected their metformin consumption status. Among the 126 patients, 60 took metformin and 66 did not. When we first performed the correlation analysis, we found a clear correlation between metformin history and *Bacteroides*-related OTUs. However, since some of these correlations were positive and others were negative, we did not conduct in-depth research on this. Previous studies on metformin and *Bacteroides* also yielded mixed results. For example, after metformin use, some studies indicated a *Bacteroides* reduction (Cherney and Lam, 2018; Sun et al., 2018), while Lee et al. (2018) reported a *Bacteroides* increase. This discrepancy may be because bacteria of the same family have opposite OTUs. Therefore, our research on gut microbiota should be accurate at the genus or species level, which is more conducive to our targeted therapy.

Hyperuric acid has an independent effect on insulin secretion in diabetic patients and plays a key role in the evolution of diabetes mellitus (DM) (Hu et al., 2018). Studies have found that elevated blood uric acid can aggravate diabetes; increase the risk of coronary heart disease, eye disease, and kidney disease; and is related to diabetes-related microvascular and macrovascular complications (De Cosmo et al., 2015; Yan et al., 2016).

Since fructose raises glucose slowly, diabetic patients usually use fructose instead of sucrose. However, studies have shown that a high fructose intake can increase uric acid and pro-inflammatory cytokine levels, intestinal permeability, and lipid accumulation in the liver and induce inflammation in the pancreas and colon (Wang et al., 2020). Our research results were consistent with these conclusions. The KEGG metabolic pathway analysis showed that the abnormal uric acid group had significantly higher fructose metabolic pathway than the normal uric acid group. Interestingly, we found that when DM patients were grouped according to their uric acid level, the BMI of the group with high uric acid was higher. Consistent with previous studies of Chen et al. (2017), this study shows that uric acid levels in diabetic patients are independently related to obesity. The research by Johnson et al. (2013) also shows that fructose-mediated uric acid production may play a causal role in diabetes and obesity.

Increased serum uric acid levels can lead to intestinal immune disorders, intestinal barrier damage, and systemic inflammation. Our results showed that the lipopolysaccharide biosynthesis function of the high uric acid group’s gut microbiota was significantly enhanced. Therefore, we speculate that gut microbiota may affect uric acid levels by regulating lipopolysaccharide synthesis. The two groups had significant differences in nucleotide metabolism, including purine metabolism and pyrimidine metabolism. This further showed that the changes in the gut microbiota of diabetic patients were associated with their serum uric acid levels.

In conclusion, our research indicated that in diabetic patients, uric acid is associated with gut microbiota, especially *Escherichia–Shigella* and *Faecalibacterium*. However, the relationship between uric acid and gut microbiota needs further exploration in non-diabetic people, including those with high uric acid levels or other diseases with high uric acid.

## Data Availability Statement

The datasets presented in this study can be found in online repositories. The names of the repository/repositories and accession number(s) can be found below: https://www.ncbi.nlm.nih.gov/, PRJNA756402.

## Ethics Statement

The studies involving human participants were reviewed and approved by The First Affiliated Hospital of Zhengzhou University Ethics Review Committee. The patients/participants provided their written informed consent to participate in this study. Written informed consent was obtained from the individual(s) for the publication of any potentially identifiable images or data included in this article.

## Author Contributions

ZZ and JS designed the study. WZ and TW carried out experiments. WZ, RG, WC, YJ, MJ, TW, WY and ZW collected samples. JS, XWa and CL analyzed the data and made the figures. JX, XWe, TW and JS drafted and revised the paper. All authors contributed to the article and approved the submitted version.

## Funding

This work was supported by the National Natural Science Foundation of China (Grant Nos. 81873611 and 8217033050), Science and Technology Innovation Team of Henan (Grant No. 17IRTSTHN020), and 2020 Key Project of Medical Science and Technology to JS.

## Conflict of Interest

CL was employed by Shanghai Mobio Biomedical Technology Co., Ltd.

The remaining authors declare that the research was conducted in the absence of any commercial or financial relationships that could be construed as a potential conflict of interest.

## Publisher’s Note

All claims expressed in this article are solely those of the authors and do not necessarily represent those of their affiliated organizations, or those of the publisher, the editors and the reviewers. Any product that may be evaluated in this article, or claim that may be made by its manufacturer, is not guaranteed or endorsed by the publisher.

## Supplementary Material

Supplementary Figure 1 Reasonable sequencing data and different fecal microbial diversity in the subgroup analysis. **(A)** Rank-abundance curves indicated that the amount of sequencing data is reasonable. Bacterial richness and diversity (including richness and evenness) were assessed by Chao index (P= 0.008; **B**) and Shannon index (P= 0.001; **C**). **(D)** NMDS analysis based on bray-curtis indicated distinct microbial community between the two groups. NMDS: non-metric multidimensional scaling.

Supplementary Figure 2
**(A–E)** Differences in gut microbiota at all levels. The N_UA and A_UA groups had significantly different relative abundances.

Supplementary Figure 3 Composition of the microbial communities of healthy controls and diabetic patients with high uric acid levels.

Supplementary Figure 4 Differential bacteria and metabolic pathways were showed after grouping according to the international hyperuricemia standard.

Supplementary Figure 5 After grouping by age, the difference in gut microbiota between DM with low uric acid and DM with high uric acid. **(A)** age <=40y, **(B)** 40y < age <55y, **(C)** age >=55y.
