# Supplementary figures and images for: Variation of Serum Uric Acid Is Associated With Gut Microbiota in Patients With Diabetes Mellitus
Source: Front Cell Infect Microbiol. 2022 Jan 18;11:761757. doi: 10.3389/fcimb.2021.761757 (PMC8803748; doi:10.3389/fcimb.2021.761757)

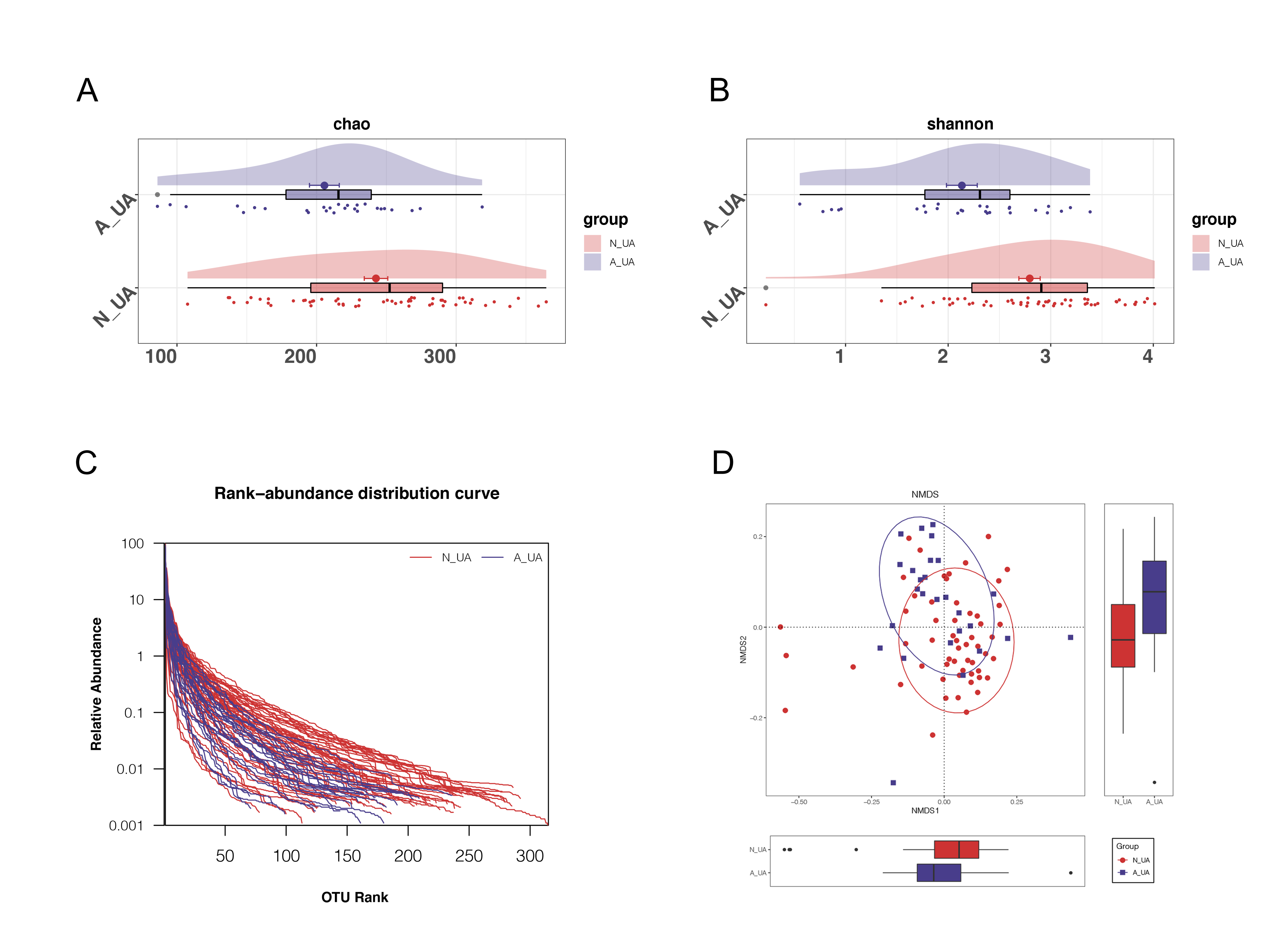

Supplement: Supplementary Figure 1 — Reasonable sequencing data and different fecal microbial diversity in the subgroup analysis. (A) Rank-abundance curves indicated that the amount of sequencing data is reasonable. Bacterial richness and diversity (including richness and evenness) were assessed by Chao index (P= 0.008; B) and Shannon index (P= 0.001; C). (D) NMDS analysis based on bray-curtis indicated distinct microbial community between the two groups. NMDS: non-metric multidimensional scaling. [file Image_1.tif]

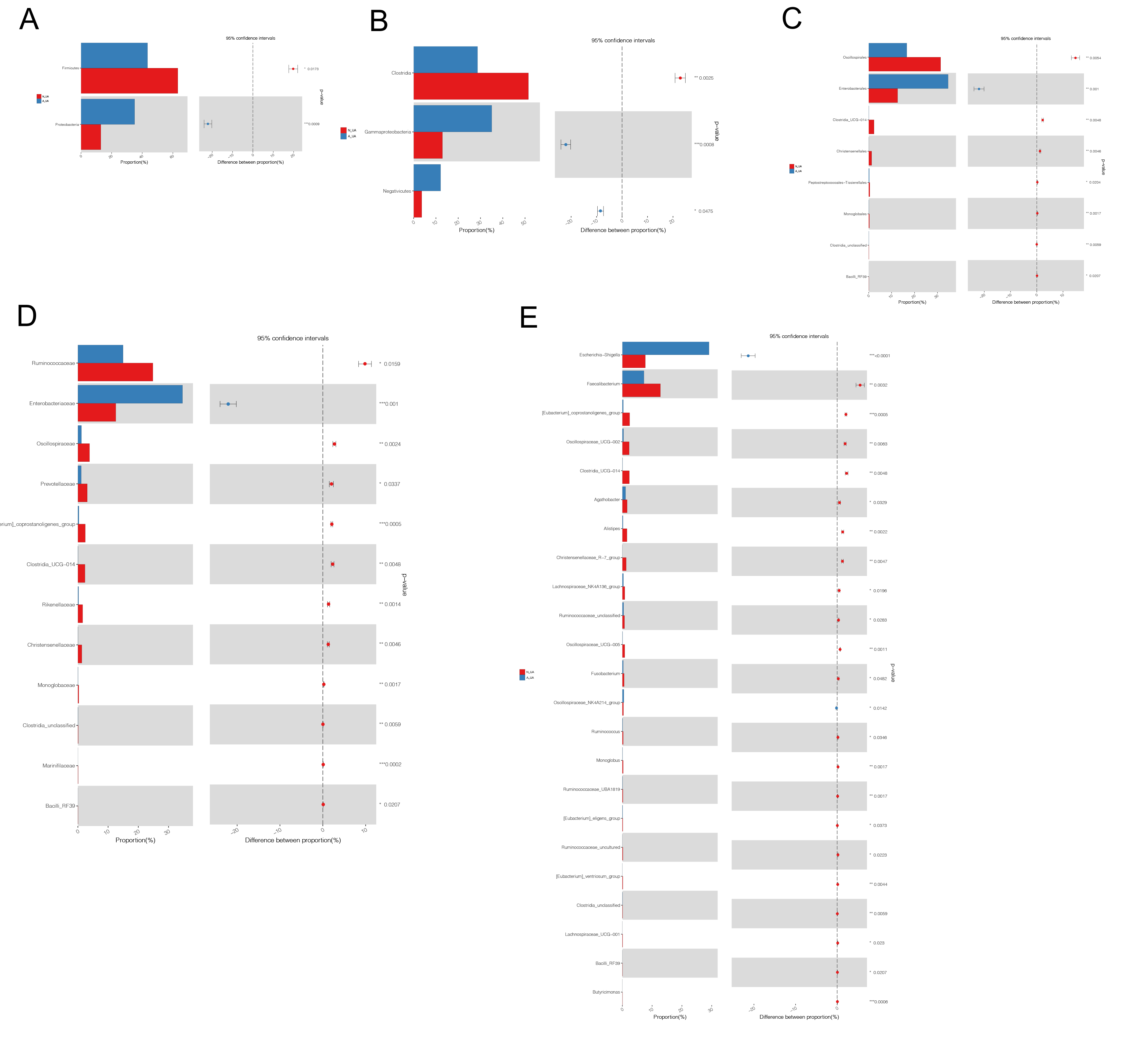

Supplement: Supplementary Figure 2 — (A–E) Differences in gut microbiota at all levels. The N_UA and A_UA groups had significantly different relative abundances. [file Image_2.tif]

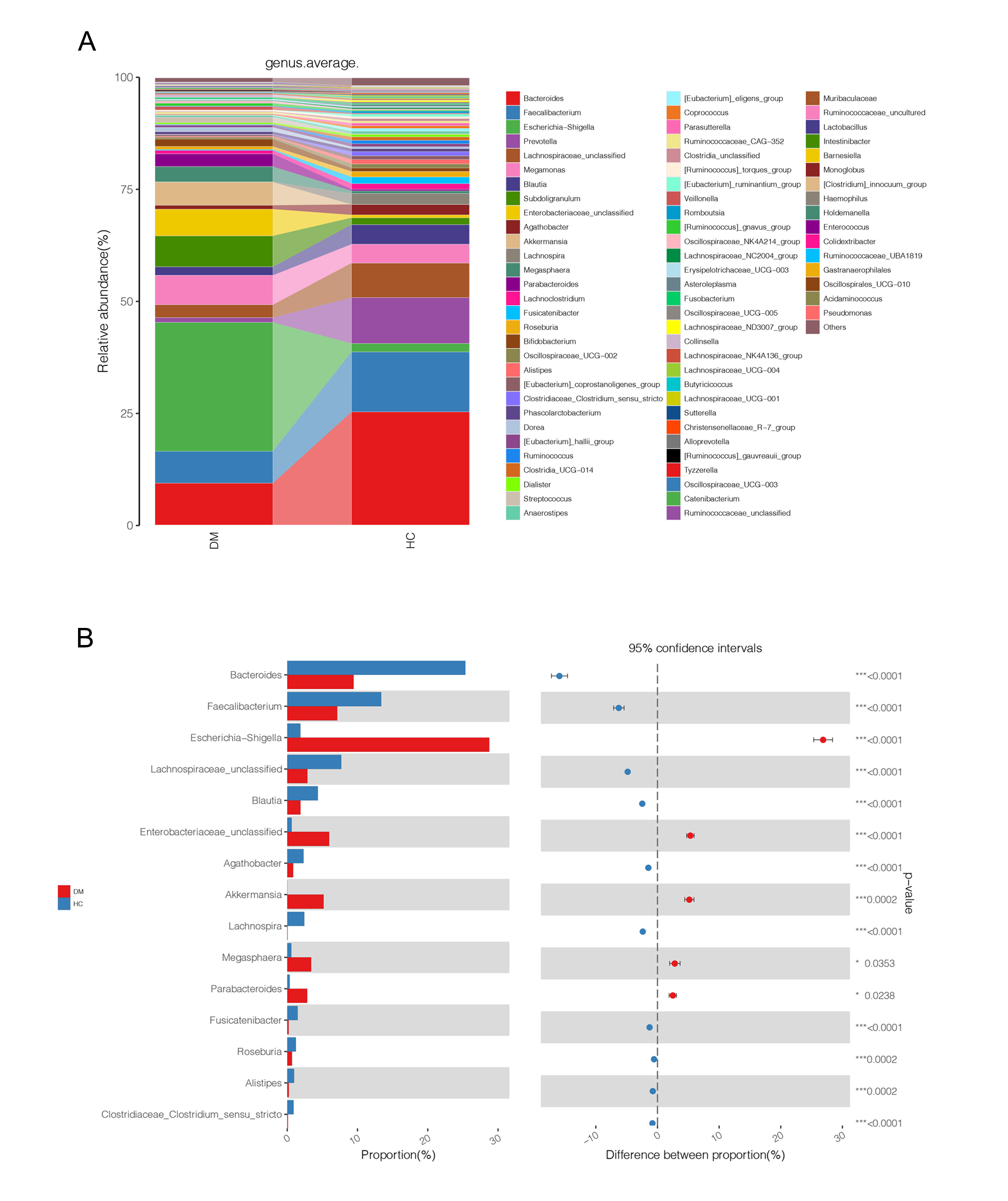

Supplement: Supplementary Figure 3 — Composition of the microbial communities of healthy controls and diabetic patients with high uric acid levels. [file Image_3.tif]

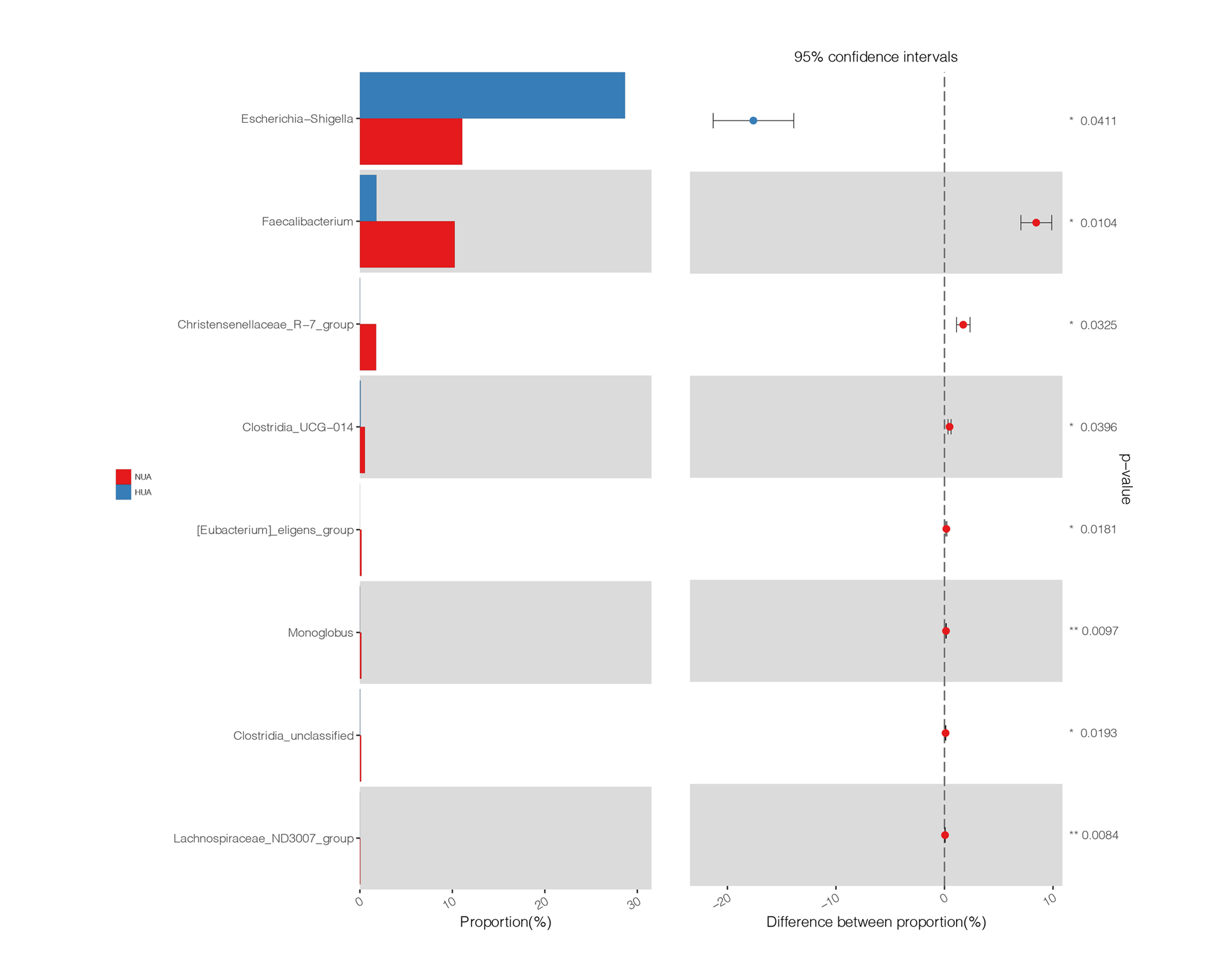

Supplement: Supplementary Figure 4 — Differential bacteria and metabolic pathways were showed after grouping according to the international hyperuricemia standard. [file Image_4.tif]

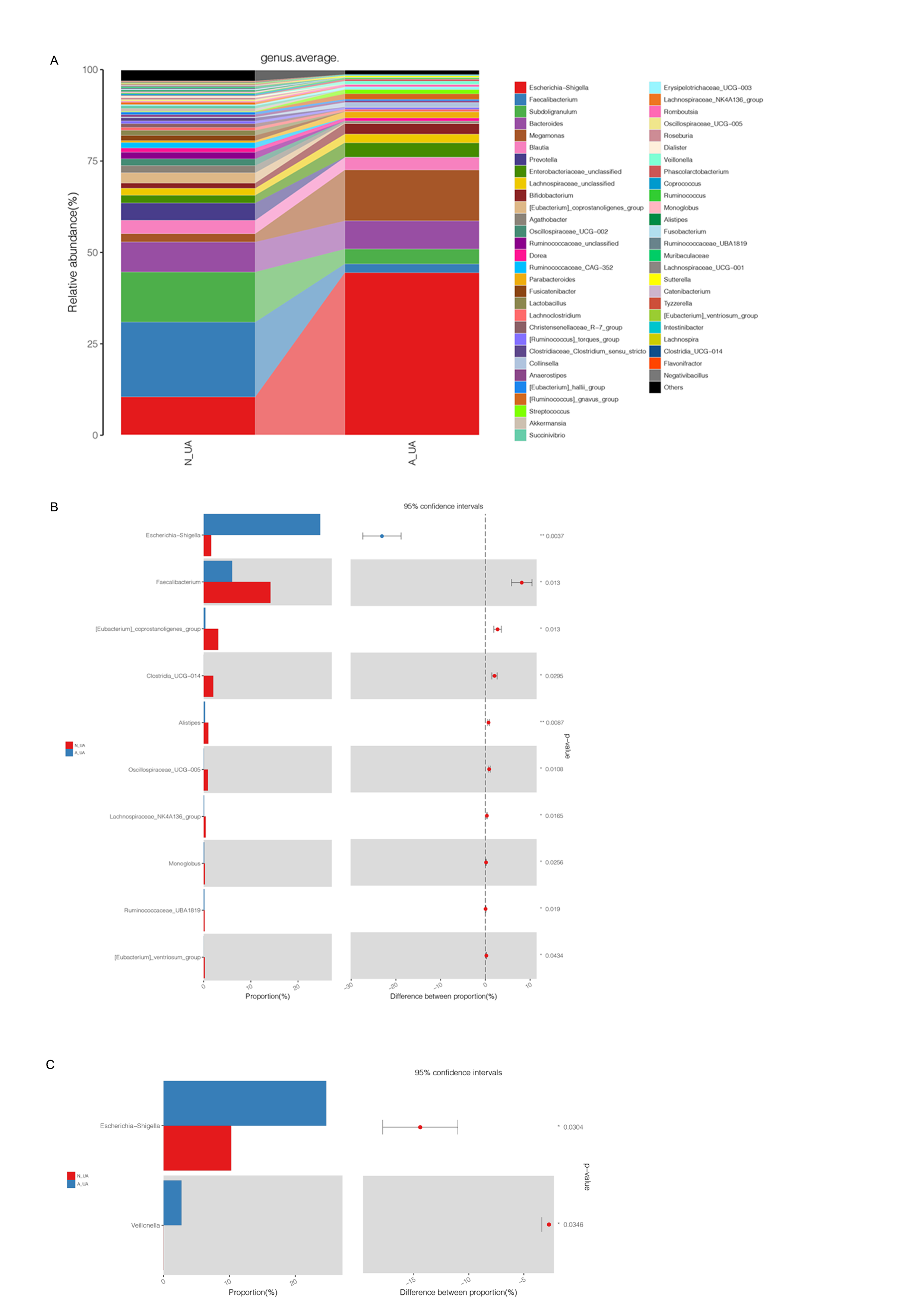

Supplement: Supplementary Figure 5 — After grouping by age, the difference in gut microbiota between DM with low uric acid and DM with high uric acid. (A) age <=40y, (B) 40y < age <55y, (C) age >=55y. [file Image_5.tif]
